# Potential roles for Kinesins at the cortical division site

**DOI:** 10.3389/fpls.2012.00158

**Published:** 2012-07-13

**Authors:** Elisabeth Lipka, Sabine Müller

**Affiliations:** Cell and Developmental Genetics, Center for Plant Molecular Biology, University of Tuebingen, Tuebingen, Germany

**Keywords:** kinesin, cell division, cortical division site, preprophase band, phragmoplast

## Abstract

Spatial control of cytokinesis is critical for cell and plant morphology. The plane of cell division is established at G2/M transition and is initially demarcated at the cortex of the cell by the cytoskeletal preprophase band (PPB) and subsequently throughout mitosis by the cortical division zone (CDZ). Few kinesins, belonging to different classes of the superfamily, either display a distinct spatio-temporal localization at the PPB and CDZ, or genetic evidence proposes a specific function there. Protein phosphorylation and degradation are likely directing the cell cycle-dependent localization and activity of some of these kinesins, as indicated by mutation of respective conserved motifs. Furthermore, kinesins are required for continuous recruitment of CDZ identity markers to the CDZ. This review summarizes the limited current knowledge of kinesins potentially involved in the steps required for correctly oriented division planes, considering localization patterns and genetic evidence, and discussing kinesin function in context with interaction partners and cell cycle regulation.

## INTRODUCTION

The cytoskeleton is a major facilitator of cell division and cell expansion in all organisms. In cellulose-enfolded plant cells, specific cytoskeletal arrays are responsible for the selection of the division plane in pre-mitotic cells and the formation of the cell plate to partition cytoplasmic contents of daughter cells during cytokinesis. The preprophase band (PPB), composed of microtubules (MTs), F-actin, and endoplasmic reticulum delineates the plane of cell division at the cell cortex. The transition from the interphase cortical MT array to the mitotic PPB involves local changes in MT dynamic behavior, regulated by the activity of MT-associated proteins (MAPs; Dhonukshe and Gadella, 2003; Vos et al., 2004). Minute detail is known about the regulation of PPB assembly; however, genes encoding MT nucleation factors TONNEAU (TON)1A and TON1B and a protein phosphatase PP2A subunit TON2/DCD1/ADD1 are strictly required for PPB formation in *Arabidopsis, Physcomitrella* and maize, respectively, since knockout mutants lack PPBs and exhibit mis-positioned division planes (Camilleri et al., 2002; Azimzadeh et al., 2008; Wright et al., 2009; Spinner et al., 2010). The PPB’s spatial information is preserved throughout mitosis by proteins, distinctly recruited to the cortical division zone (CDZ), formerly occupied by the PPB, and by proteins selectively depleted from the CDZ. Thus, the CDZ is tagged by positive and negative identity markers. Progressive confinement of the CDZ during cytokinesis specifies the precise site of cell plate fusion, the cortical division site (CDS).

Among the 61 predicted kinesins in *Arabidopsis*, only about one-third were up-regulated during mitosis (Menges et al., 2003; Lee and Liu, 2004; Vanstraelen et al., 2006a) and even fewer were implicated in division plane selection and maintenance (Zhu and Dixit, 2011).

## DIVISION PLANE SELECTION AND PPB FORMATION

The position of the nucleus is informative for division plane orientation (Muller, 2011; Rasmussen et al., 2011a). Displacement of the prophase nucleus in protonemata leads to the formation of a new PPB, encircling the dislodged nucleus (Murata and Wada, 1991). Prior to proliferative, symmetric divisions in pre-mitotic cells, the nucleus is centered presumably by MT length-dependent forces (Goodbody et al., 1991; Besson and Dumais, 2011).

Recently, members of the KCH subgroup of kinesin-14 class were implicated in nuclear migration. A number of KCH proteins tested so far displayed actin binding activity conferred by the conserved Calponin homology (CH) domain (**Figure 2**) potentially linking the MT and the actin cytoskeleton (Frey et al., 2009; Buschmann et al., 2011; Klotz and Nick, 2012). A study revealed the existence of motile, MT-associated NtKCH populations at the cell cortex, and non-motile peri-nuclear populations associating with actin in interphase of tobacco BY-2 cells (Klotz and Nick, 2012). Consistent with overexpression of its *Arabidopsis* homolog AtKinG in BY-2 cells and with other kinesins in that class, NtKCH motility was MT minus end directed (Lee and Liu, 2004; Buschmann et al., 2011). Furthermore, motile NtKCH associated with a subset of MTs, bridging the nucleus with the cell cortex. Thus, it was suggested that KCH might act in the positioning of the nucleus involving a combination of MT dynamics and actin anchored KCH sliding toward MT minus ends (Klotz and Nick, 2012). Indeed, pre-mitotic nuclear migration was significantly delayed in tobacco BY-2 cells overexpressing GFP-KCH1 from rice (OsKCH1; Frey et al., 2010).

Kinesin-14 class members ATK1 and KCBP and kinesin-5 AtKRP125c co-localized with the PPB, but also with interphase and mitotic MT arrays supporting a more general role in MT bundling (Bowser and Reddy, 1997; Marcus et al., 2003; Bannigan et al., 2007). Although *atk1* mutants display wider PPBs indicating a role in PPB formation, an impact on the CDZ or cell wall positioning was not reported (Marcus et al., 2003).

**FIGURE 1:**
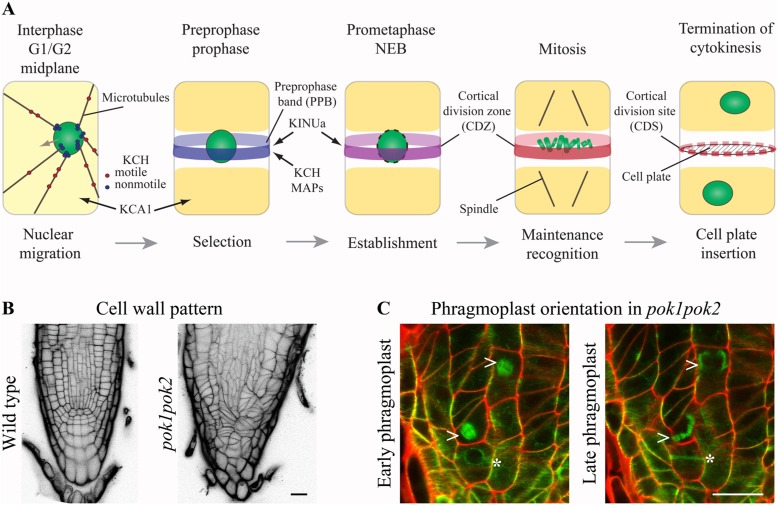
**Kinesins at the cortical division zone (CDZ)**. **(A)** In pre-mitotic cells, nuclear migration to the cell center is accomplished by microtubule (MT) force-dependent sensing of cell geometry potentially mediated by KCH kinesins. The preprophase band (PPB) circumscribes the future division plane at the cell cortex. Several MT-associated proteins (MAPs) and kinesin AtKINUa and the *Arabidopsis* KCH (AtKinG) are recruited to the PPB/CDZ, while the plasma membrane resident kinesin KCA1 becomes depleted from this site. Nucleus/chromosomes are indicated in green. **(B** and **C)** Confocal micrographs of seedling root tips. **(B)** Wild type and *pok1pok2* cell wall patterns, visualized by Propidium Iodide (PI) staining. **(C)** Change of phragmoplast (arrow head) orientation from a transverse to orthogonal plane in *pok1pok2*. Asterisk indicates PPB. Green label: GFP-MT marker, red label: cell wall by PI staining. Scale bar 20 μm.

The function of the negative CDZ marker, kinesin KCA1 remains enigmatic (Vanstraelen et al., 2006b). KCA1 accumulated at the plasma membrane at high levels during mitosis, but remained absent from the CDZ, presenting a KCA1 depleted site and resembling aspects of F-actin distribution (**Figure 1**). As indicated by MT-depolymerization experiments, formation of the KCA1 depleted site depended on prior PPB assembly and absence of the KCA1 depleted site lead to mis-positioning of cell plates. Similarly, drug induced depolymerization of F-actin before formation of the actin depleted zone/microfilament twin peaks (ADZ/MFTP) disturbed proper cell plate positioning in BY-2 cells (Hoshino et al., 2003; Sano et al., 2005). In contrast, KCA1 localization did not alter upon actin depolymerization (Vanstraelen et al., 2006b). Interestingly, *kca1kca2* double mutants were defective in light-induced chloroplast movement (Suetsugu et al., 2010), a process known to be actin dependent. It is likely that the CDZ requires an environment of reduced motility to recruit and maintain a certain suite of proteins (Panteris, 2008). Experiments pertaining to the temporal relations between KCA1 and other CDZ markers should help elucidate the significance of differential KCA1 localization.

KCA1 and its homolog KCA2 were initially identified as CDKA;1 interaction partners (Vanstraelen et al., 2004). The KCAs shared a conserved domain structure with N-terminal motor domain. However, the motor domain was most similar to that of C-terminal kinesins and preceded by a neck-linker and therefore, KCAs were placed within the kinesin-14 subfamily (Vanstraelen et al., 2004). The subsequent stalk domain-mediated homo- and hetero-dimerization *in vitro* (Vanstraelen et al., 2004) and the C-terminal tails of KCA1 and KCA2 featured three and two CDKA;1 phosphorylation sites, respectively. Site directed mutagenesis indeed reduced binding to CDKA;1 *in vitro* and intra-molecular folding of the tail onto the stalk was obstructed. Thus, KCA activity might be regulated depending on their phosphorylation status, which might be addressed by expression of phospho-mimic mutants in plants.

Progression through the cell cycle depends on the timely degradation of cell cycle regulatory proteins, to ensure synchronization of chromatin condensation and mitotic cytoskeletal array formation. Recently, the kinesin AtKINUa/ARK3 was proposed to act as a synchronizer (Sakai et al., 2008; Malcos and Cyr, 2011). AtKINUa is a member of a small ungrouped class of kinesins, present in plants and protists. Their domain structure is unique, comprising a non-conserved N-terminal motor domain and a variable number of armadillo repeats at their C-terminus, however, lacking a characteristic neck-linker (Malcos and Cyr, 2011). Furthermore, these kinesins contain a conserved destruction box (D-BOX) motif, serving as a potential target for proteasome-mediated degradation. AtKINUa associated with cortical MTs in interphase, but became highly enriched at the PPB in prophase and eventually disappeared upon nuclear envelope breakdown (NEB) in metaphase. Intriguingly, not only AtKINUa degradation at the NEB depended on the D-BOX motif, but also the protein’s association with PPB MTs. The mutation of a conserved residue within the D-BOX motif resulted in diffuse accumulation of AtKINUa-GFP at the PPB, as well as at the spindle and phragmoplast, indicating that timely degradation of the fusion protein was obstructed (Malcos and Cyr, 2011). Immediately succeeding the D-BOX is a putative CDKA phosphorylation site, however, its significance for AtKINUa localization was not evaluated so far. AtKINUa was distinctly expressed in embryos and cells of the stomatal lineage in *Arabidopsis*, but, genetic evidence for specific function of AtKINUa during cell division is not available yet (Sakai et al., 2008; Malcos and Cyr, 2011).

The confined spatio-temporal localization pattern of AtKINUa and its protein domains narrow the number of potential interaction partners to several PPB-associated MAPs such as MOR1, CLASP, AIR9, or MAP65s (Buschmann et al., 2006; Kawamura et al., 2006; Ambrose et al., 2007) and above mentioned kinesins ATK1, KCBP, and AtKRP125c. TON2/DCD1, however, showed a spatio-temporal distribution very similar to AtKINUa (Wright et al., 2009) making it a probable interaction partner. Indeed, recently shown genetic interaction between *TON2* and *MOR1* and *TON2* and *TON1* further validated the involvement of these proteins in division plane placement (Kirik et al., 2012). Most likely, TON2-dependent dephosphorylation modulates the activity of MOR1 and TON1 in MT array organization. Thus, functional aspects of AtKINUa might be revealed by investigating localization of endogenous and AtKINUa phospho-mutants in mutants defective in PPB formation and cell wall positioning.

## DIVISION PLANE MAINTENANCE

**FIGURE 2:**
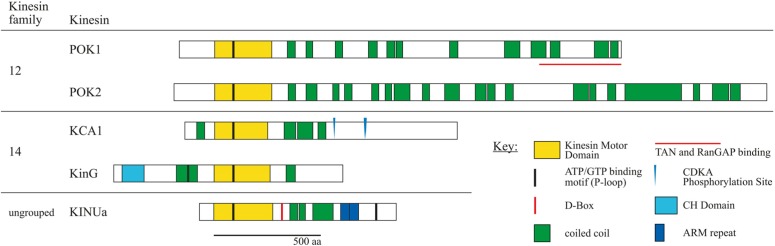
**Schematic overview of kinesin proteins implicated in cortical division site (CDS) establishment by experimental evidence**. Subfamily affiliations are according to the nomenclature (Lawrence et al., 2004). Proteins were co-aligned with respect to the P-loop motifs within the motor domain. Experimentally confirmed D-BOX motifs and CDKA phosphorylation sites are indicated. Amino acid sequences were obtained from TAIR. Kinesin motor domains, P-loop motifs, armadillo (ARM) repeats, and calponin homology (CH) domains were predicted using ScanProsite tool at Expasy (http://prosite.expasy.org/scanprosite). Coiled coil domains were predicted using Parcoil2 at MIT, with a cut off value *P* > 0.025 and minimum read of 28 (http://groups.csail.mit.edu/cb/paircoil2). Proteins are drawn to scale.

PHRAGMOPLAST ORIENTING KINESIN (POK) 1 and 2 were required for the preservation and/or recognition of spatial information conveyed by the PPB. POKs belonged to the kinesin-12 class based on their N-terminal motor domain and were the largest predicted kinesins in *Arabidopsis* (**Figure 2**; Lee and Liu, 2004). Despite their significant size difference (**Figure 2**) and overlapping, yet distinct gene expression patterns, POK1 and POK2 exhibited functional redundancy. While single mutants were indistinguishable from wild-type, double mutants of T-DNA insertion alleles displayed dwarfed overall morphology and pronounced mis-orientation of cell walls in root meristems, deviating from the regular pattern characteristic for wild-type (**Figure 1B**; Muller et al., 2006).

The plant-specific MAP TANGLED and the Ran regulatory protein RanGAP1, both positive CDZ identity markers, co-localized with the PPB and the CDZ throughout mitosis. TAN as well as RanGAP1 fusion proteins were inadequately recruited to the PPB in *pok1pok2* (Walker et al., 2007; Xu et al., 2008). Moreover, TAN and RanGAP1 association with the CDZ was not maintained past metaphase, revealing the dynamic nature of the CDZ. Strikingly, *pok1pok2* phragmoplasts appeared to lack guidance (**Figure 1C**) and the cell plate fused with the parental cell wall seemingly random, wherever they encountered upon completion of cytokinesis. Since the initial recruitment of TAN and RanGAP1 to the PPB occurred independent of POKs (Xu et al., 2008; Rasmussen et al., 2011b), kinesins other than POK1 and POK2 might support this task. The C-terminus of POK1-mediated interaction with both, TAN and RanGAP1 (Muller et al., 2006; Xu et al., 2008), suggesting that POK1 itself localized to the CDZ.

## CONCLUDING REMARKS

KCAs and AtKINUa are documented instances for the impact of cell cycle-dependent phospho-regulation and protein degradation on protein activity and localization. Notably, the CDK consensus motif [S/T-P-x-K/R] was detected several times in POKs, in AtKinG (KCH) and a phosphorylation site was predicted for AtKINUa (Malcos and Cyr, 2011). Furthermore, consensus D-BOX motifs [R-x_2_-L-x_4_-N/Q] were present in POKs (manual annotation and Vanstraelen et al., 2006a).

So far, motility was only reported for KCH kinesins. Future research assignments certainly involve *in vivo* live-cell imaging studies as well as *in vitro* assembly and imaging to investigate kinetics of kinesins taking part in CDZ selection and maintenance. However, successful analysis depends on the availability of functional fusion proteins which may be confirmed by complementation of mutants or consensus localization with specific antibodies. Furthermore, the low abundance of these kinesins poses challenges, even for sensitive imaging systems. Nevertheless, recent technical advances in imaging may contribute to tackle these objectives.

## Conflict of Interest Statement

The authors declare that the research was conducted in the absence of any commercial or financial relationships that could be construed as a potential conflict of interest.
